# Correspondence

**Published:** 1875-12

**Authors:** Edmund J. Doering

**Affiliations:** Asst. Surg. U. S. M. H. S.; San Francisco


### Correspondence.

San Francisco, Oct. 14, 1875.

Editor Chicago Medical Journal and Examiner—

Dear Sir:—Twenty-two seamen, affected with scurvy, in a more or less severe form, were recently admitted from the British Ship *Bremen* into the U. S. Marine Hospital at this port. That such a preventable disease as scurvy should still occur, notwithstanding the Act of 1872, which requires vessels, crossing the ocean, to be provided with lime-juice and other anti-scorbutics, clearly points to the necessity of further legislation on this subject, as suggested by Dr. John M. Woodworth, the Supervising Surgeon-General of the U. S. Marine Hospital Service, in his last annual report. In view of these facts, the general history of these cases may be of some interest. The *Bremen*, formerly engaged as a steamer between Bremen and New York, but at present transformed into a clipper ship, is 328 feet long, 40 feet beam, 26 feet deep, and registers 2,794 tons. She is in command of Captain Lawrence S. Leslie, and is owned by Edward Bates, M. P. Mr. Plimsoll, in his recent extraordinary address before the House of Commons, referred to Bates when he spoke of the ship-owners outside of the House and their "confederates within," and gave notice of a question to be put to Mr. Bates to explain the loss of six of his vessels in 1874, and then very touchingly added: "I am determined to unmask the villains who send their sailors to death." The following statements are essentially those given by the captain before a Naval Court of Inquiry, which investigated the case after the arrival of the ship in port.

On the 6th of February the *Bremen* sailed from Liverpool for this port with a cargo of coal, the crew consisting of the captain, three mates, carpenter, sailmaker, apprentices, one white seaman and thirty-six colored seamen. She was well provisioned in every respect, and sailed with every prospect of a fair voyage. On February 12th, six days after leaving port, the first case of sickness was reported to the captain, the invalid being Charles Parry, one of the colored crew, who complained of a bad chest and throat. The captain, after an examination, found that his lungs were completely gone, which marvelous discovery induced him to prescribe at once a dose of cough-pills. The poor patient, however, was unable to swallow, and in consequence died a few days later.

From this time on, sickness continued to increase among the crew, although the captain commenced to serve out lime-juice on Feb. 14th. Symptoms of scurvy began to appear in March, and the captain puts the blame on the sailors, in explanation of the fact that so many of the crew were taken down with this loathsome disease. According to his statement, when sick, they would refuse to take the vinegar and other medicines prescribed, until they were helpless. They also became demoralized and frightened on seeing their number diminishing one by one. Another feature was, that as soon as the ship came out of cold weather into warm, the black men went down "like sunflowers."

On April 25th the ship was off Cape Horn, and on April 28th the captain ordered fresh messes to be served out twice a week, instead of twice a month. On May 7th the forecastle was disinfected. Still the disease continued and reached an epidemic form, until three-fourths of the crew were helpless. On May 21st the sick men were removed from the forecastle into an improvised hospital on the main deck. The food consisted of sago, rice, arrowroot, oatmeal, and fresh meat twice a week. Alum gargle was ordered for the mouth and quinine and wine given internally. June 3rd the captain ordered three times the ordinary quantity of lime-juice to be given to the crew, as he had an abundant supply on board, but many of the men refused to take it. On his official log-book the captain has an entry that some penalty should be attached to seamen who refuse to take lime-juice and eat good pressed vegetables.

Thirteen of the crew died during the voyage. The last death occurred two days before the ship arrived in San Francisco, which port was finally reached after a tedious voyage of 191 days. It ought to be added in this connection that on one occasion when Mr. Plimsoll called attention to the ships of Mr. Bates, and spoke of the carelessness with which the wants of the seamen were looked after, he made particular mention of the *Bremen*, and stigmatized her as a "floating coffin." His statement has been verified to the letter.

The story as told by the seamen, some of whom are more than usually intelligent, differs in some respects from that given above. According to their uniform statement, the meat was very bad, hardly fit to eat; the supply of vegetables insufficient and poor, and the usual ration of bread reduced to one-half, after they passed Cape Horn.

The Court of Inquiry investigated the case very thoroughly, and reported adversely to the captain, censuring him for not having called in at a way port, to procure a supply of fresh vegetables. This is another proof that "the invariable and indispensable antecedent of scurvy has been a deficiency or absolute want of fresh vegetable food."

On admittance to the hospital, the men presented the usual symptoms of scurvy; general depression of the physical powers; pain in the limbs, with hardening of the muscles, especially the hamstring muscles, from fibrinous deposit; swelling, sponginess and bleeding of the gums, with a blue line along the edge; offensive breath, etc.

The treatment consisted in supplying the patients with plenty of beef tea, and giving them three grs. of the citrate of iron and quinine, three times a day, with a solution of carbolic acid for the mouth. Under this treatment, with careful nursing and a well regulated diet, the patients rapidly recovered, only one case resulting fatally, due to general exhaustion.

EDMUND J. DOERING, M.D.,
Asst. Surg. U. S. M. H. S.

